# The effect of sleep pattern changes on postpartum depressive symptoms

**DOI:** 10.1186/s12905-017-0496-6

**Published:** 2018-01-09

**Authors:** Beth A. Lewis, Dwenda Gjerdingen, Katie Schuver, Melissa Avery, Bess H. Marcus

**Affiliations:** 1 School of Kinesiology, University of Minnesota, 1900 University Ave SE, Minneapolis, MN 55455 USA; 2 Department of Family Medicine & Community Health, University of Minnesota, Minneapolis, MN 55455 USA; 3 School of Nursing, University of Minnesota, 308 Harvard Street SE, Minneapolis, MN 55455 USA; 4 Department of Family and Preventive Medicine, University of California, San Diego, 9500 Gilman Drive, 0628, La Jolla, CA 92093 USA

**Keywords:** Postpartum depression, Sleep, Exercise, Physical activity

## Abstract

**Background:**

Research indicates that poor sleep is associated with postpartum depression; however, little is known regarding this relationship among postpartum women who are at high for postpartum depression. This study examined the relationship between changes in self-reported sleep patterns (from six weeks to seven months postpartum) and depressive symptoms at seven months postpartum among women who were at high risk for postpartum depression.

**Methods:**

Participants (*n* = 122) were postpartum women who were at an increased risk for postpartum depression (personal or maternal history of depression) and had participated in a randomized exercise intervention trial. For the current trial, participants completed the Pittsburgh Sleep Quality Index and Patient Health Questionnaire-9 (PHQ-9; assessed depression) at six weeks and seven months postpartum.

**Results:**

Overall, sleep problems significantly improved from six weeks to seven months postpartum. However, linear regression analyses indicated that worsening or minimal improvement of sleep problems were associated with higher depressive symptoms at seven month postpartum. Regarding the specific types of sleep problems, self-reported changes in sleep latency (i.e., how long it takes to fall asleep at night), daytime dysfunction (i.e., difficulty staying awake during the day), and sleep quality (i.e., subjective rating of sleep quality) were associated with higher levels of depressive symptoms.

**Conclusions:**

Sleep problems typically improve during the postpartum phase. However, postpartum women who are at high risk for postpartum depression are at an increased risk for depressive symptoms later in the postpartum phase if sleep problems worsen or show only minimal improvement over time. Therefore, at the six-week postpartum clinic visit, women should receive education regarding potential worsening of sleep patterns and strategies for preventing sleep-related problems.

**Trial registration:**

Registered with ClinicalTrials.gov (NCT00961402) on August 18, 2009 prior to the start of the trial.

## Background

Postpartum depression is associated with numerous maternal and infant-related consequences including poor infant-child bonding [1], difficulty caring for the newborn [2], long-term behavior problems for the child [3], more weight retention for the mother [4], and future depression risk for both parents [5]. This is problematic given approximately 13–19% of mothers experience postpartum depression [6–9]. Among women who have a history of depression, 31% report depression during pregnancy and/or postpartum [10, 11]. Additionally, research indicates that approximately one-quarter of mothers report depressive symptoms but do not meet the full criteria for depression [12].

Sleep is significantly disrupted during the postpartum period and therefore, may play an important role in the development of depression [13]. Poor sleep can continue through 12 months following the birth of a baby and beyond [14, 15]. Lack of sleep can result in exhaustion, impatience, lower ability to concentrate, and a poor quality of life [16], which can all contribute to an increased risk for postpartum depression.

Recent reviews indicate that sleep problems are related to postpartum depression [13, 17]. However, more research is needed regarding the long-term effect of poor sleep on postpartum depression. For example, several studies link poor sleep to postpartum depression but some have examined postpartum depression at less than four weeks postpartum [18–20]. Additionally, several studies examined the correlation between depression and sleep at the same time points, even when assessing both sleep and depression at multiple time points in some studies. Therefore, the influence of poor sleep over time on the development of depression was not examined in these studies [21–26].

Given poor sleep is a diagnostic criteria for depression based on the DSM-V (American Psychiatric Association APA; [27]), it is important to examine poor sleep and depression at separate time points in order to identify a potential causal link between changes in sleep and the onset of postpartum depression. Longer-term studies have found that sleep problems in pregnancy and early in the postpartum period are related to higher depressive symptoms at 17–36 weeks postpartum [28, 29]. As an example, Okun and colleagues [30] conducted a longitudinal study examining the effect of poor sleep on depression among postpartum women enrolled in a randomized trial examining the efficacy of an antidepressant medication. Participants completed the Pittsburgh Sleep Quality Index PSQI; [31] and the Hamilton Rating Scale for Depression HRSD; [32] during the first 17 weeks postpartum. Results indicated that increases on the PSQI (indicating poorer sleep) were related to a higher rate of depression. One limitation of this study is that over half of the participants were administered an antidepressant during the study, although this was controlled for in the analysis. This study lacked long-term follow-up past 17 weeks postpartum. Additionally, it is unclear which particular type of sleep problem (e.g., nighttime awakenings, difficulty falling asleep) accounted for the effect of poor sleep on depression.

The purpose of this study was to address the limitations of previous studies. First, we assessed prospective associations between changes in postpartum self-reported sleep problems and depressive symptoms during the postpartum period. Second, we examined which types of sleep problems were associated with postpartum depressive symptoms. Finally, we examined women who were at an increased risk for postpartum depression (84% had a personal history of depression and 16% had a maternal history of depression), which has not been explored previously to our knowledge. This is a particularly important group to examine given women with a history of depression are 2–3 times more likely to experience postpartum depression than women without a history of depression [10, 11].

The current study examined the relationship between changes in self-reported sleep patterns (from six weeks to seven months postpartum) and depressive symptoms at seven months postpartum. Specifically, we predicted that worsening of self-reported sleep problems from six weeks to seven months postpartum would be related to increased depressive symptoms at seven months postpartum. We also explored the relationship between depressive symptoms and changes in specific self-reported sleep problems including sleep duration (i.e., amount of nighttime sleep), sleep disturbance (i.e., number of awakenings during the night), sleep latency (i.e., how long it takes to fall asleep at night), daytime dysfunction (i.e., difficulty staying awake during the day), sleep efficiency (i.e., percentage of time asleep when in bed), sleep quality, and the use of sleep medications. Breastfeeding was controlled for in the analyses given its association with increased nighttime awakenings [33].

## Methods

### Participants

Participants (*n* = 122) were healthy women from the upper Midwest in the United States who were on average six weeks postpartum at the first assessment. They were enrolled in a randomized trial examining the effect of a telephone-based exercise intervention on preventing postpartum depression. In order to recruit participants who were at high risk for postpartum depression, participants had a history of depression and/or their mother had a history of depression (84% had a personal history of depression). Participants were recruited via a local parent magazine, Craig’s list, and targeted emails.

Exclusion criteria were assessed using a telephone screening interview, which was administered either during pregnancy or at less than six weeks postpartum. Exclusion criteria included the following: (1) Less than 18 years old; (2) exercising more than 60 min per week; (3) participating in another exercise-related study; (4) does not speak English; (5) another person in the household taking part in the study; (6) could not walk for 30 continuous minutes before pregnancy; (7) psychiatric-related hospitalization during the past six months; (8) pre-existing hypertension or diabetes; (9) musculoskeletal problems that may interfere with exercising; (10) taking medication that changes heart rate response to exercise; (11) exercise-induced asthma; (12) current depressive episode; and (13) any condition that would make exercise unsafe. This trial was approved by the Institutional Review Board at the University of Minnesota.

### Measures

Demographic variables were assessed during both the telephone screening interview (race-ethnicity, age) and via a questionnaire sent through the mail (marital status, income, education level, and number of children at home).

#### The Pittsburgh sleep quality index (PSQI)

Sleep was assessed using the 19-item PSQI, which is a self-report questionnaire that assesses sleep quality and disturbances during the previous month [31]. The seven component subscales include subjective sleep quality (i.e., subjective self-rating of sleep quality), sleep latency (i.e., how long it takes to fall asleep at night), sleep duration (i.e., amount of nighttime sleep), habitual sleep efficiency (i.e., percentage of time asleep when in bed), sleep disturbances (i.e., number of awakenings during the night), use of sleep medication, and daytime dysfunction (i.e., difficulty staying awake during the day). This scale has good internal consistency, test-retest reliability, and validity [31]. The diagnostic sensitivity and specificity were 89.6% and 86.5% respectively in one study among adults with and without sleep disorders [31]. Several studies have used the PSQI with postpartum women [17, 29, 30].

#### The patient health Questionnaire-9 (PHQ-9)

The PHQ-9 was used to assess depressive symptoms. This scale evaluates nine symptoms of depression based on the DSM-IV and each item is rated on a Likert scale ranging from 0 to 3 [34]. Specific items include diminished pleasure, depressed mood, sleep difficulty, low energy, appetite/eating changes, self-deprecation, difficulty concentrating, psychomotor changes, and suicidal thoughts. This scale has been found to have high sensitivity (73–88%) and specificity (88–98%) for identifying major depressive disorder. Additionally, the PHQ-9 has been recommended as a tool for diagnosing depression [35–37], identifying subthreshold depressive disorders [37], and evaluating depression outcomes [38–40].

### Procedure

In the overall trial, interested and eligible participants (based on the telephone screening interview) were mailed consent forms and their healthcare providers were faxed provider consent forms. Once participants returned their consent form and demographic questionnaire via the mail and healthcare provider consent forms were received, participants (*n* = 130) were randomly assigned to a telephone-based exercise intervention or a telephone-based wellness control condition, both lasting six months. Participants were randomized on average at six weeks postpartum, which was when the first assessment occurred for the current study. Participants who were depressed at baseline based on the Structured Clinical Interview for DSM-IV Axis I Disorders SCID-I; [40] were excluded from the study (*n* = 1). The design and results of the overall trial are described in more detail elsewhere [41, 42]. In sum, there were no differences between the two study conditions on depression at seven months postpartum. Specifically, 8% from each group met the diagnostic criteria for depression based on the Structured Clinical Interview for DSM-IV Axis I Disorders SCID-I; [40]. Participants in both conditions exercised at similar rates (128 min for the exercise condition and 122 min for the wellness condition).

For the current study, participants completed the Pittsburgh Sleep Quality Index to assess sleep quality at six weeks and seven months postpartum. Participants also completed the Patient Health Questionnaire-9 (PHQ-9) at both six weeks and seven months postpartum. Eight participants who completed the baseline questionnaires did not complete the seven month questionnaires resulting in a final sample size of *n* = 122 for the current study.

### Data analysis

We calculated change scores by subtracting sleep scores at six weeks postpartum from sleep scores at seven months postpartum. We then used linear regression to examine the relationship between change in sleep from six weeks to seven months postpartum on depressive symptoms at seven months after controlling for six week depressive symptoms, breastfeeding at birth, and condition assignment (exercise vs. wellness control condition). SPSS was the statistical package used to conduct the data analysis. The cut-off for determining significance was *p* < .05.

## Results

### Summary of sample

The majority of the participants were married (82%), Caucasian (82%), college educated (69%), had an annual income of over $50 k (62%), and most had other children (76%). Sixty-six of the participants had been randomized to the exercise condition and 64 to the wellness control. There was no differential drop-out between conditions (92% of the exercise arm and 98% of the wellness control arm completed the 6-month follow-up). Condition assignment was controlled for in all of the analyses.

### Sleep index and PHQ-9 summary

Means scores on the PHQ-9 (i.e., depressive symptoms) were 5.98 (*sd* = 3.83) at six weeks and 4.17 (*sd* = 4.04) at seven months postpartum. Scores on the PHQ-9 significantly decreased from six weeks to seven months postpartum, *f*(1, 124) = 18.93, *p* < .001. The overall global score and subscale scores on the Pittsburgh Sleep Quality Index are presented in Table 1. Global scores on the PSQI significantly decreased from six weeks to seven months postpartum, *f*(1, 122) = 34.19, *p* < .001 indicating improved sleep over time.

**Table 1:** Pittsburgh Sleep Quality Index (PSQI) Mean Scores at Each Timepoint

Variable	Six Weeks (*n* = 130)	Seven Months (*n* = 122)
Global	7.11 (2.84)	5.53 (3.09)
Duration	1.02 (0.98)	0.66 (0.88)
Disturbance	1.12 (0.50)	1.02 (0.48)
Latency	0.79 (0.85)	0.73 (0.91)
Daytime Dysfunction	1.15 (0.85)	0.87 (0.75)
Efficiency	1.55 (1.14)	0.82 (0.99)
Quality	1.30 (0.67)	1.22 (0.74)
Medication Use	0.17 (0.59)	0.22 (0.65)

Higher scores represent worsening sleep. Standard deviations are in parentheses

### Relationship between change in self-reported sleep and depressive symptoms

The relationship between changes on the Pittsburgh Sleep Quality Index Overall Score (PSQI) total score from six weeks to seven months postpartum and depressive symptoms at seven months was examined. Change scores were calculated by subtracting sleep scores at six weeks postpartum from sleep scores at seven months postpartum. Additionally, the sleep item on the PHQ-9 was removed for this analysis at both six weeks and seven months to avoid overlap with the PSQI. Even though omitting this item does attenuate the reliability and validity of this scale, it is necessary to omit the item given the direct overlap between this sleep item and the PSQI. Linear regression analyses indicated that after controlling for depressive symptoms at six weeks postpartum, breastfeeding at birth, and condition assignment, greater increases from six weeks to seven months postpartum on the PSQI (higher scores indicate poorer sleep) predicted higher depressive symptoms at seven months postpartum (see Table 2). To examine which subscales of the PSQI accounted for the effect, additional linear regression analyses were conducted for each of the sleep index subscales. Specifically, greater increases (indicating poorer self-reported sleep and functioning) from six weeks to seven months postpartum on daytime dysfunction, sleep latency, and sleep quality subscales predicted higher depressive symptoms at seven months postpartum (see Table 3). However, changes in the sleep duration, sleep disturbance, sleep efficiency, and needing medication to sleep subscales from six weeks to seven months postpartum did not predict depressive symptoms at seven months postpartum. To test for multicollinearity, the variance inflation factors (VIF) were calculated for each of the predictor variables in the regression analyses. A value of five or greater is indicative of multicollinearity [43]. The variance inflation factors ranged from 1.002 to 1.056 for the predictors in the regression analyses, indicating low multicollinearity.

**Table 2:** Change in Overall Sleep Score from Six Weeks to Seven Months Postpartum Predicting Seven Month Depressive Symptoms

Variable	Beta^a^	SE	*P*-value
Baseline PHQ-9 (Depressive Symptoms)^b^	0.367	0.083	.000
Breastfed at Birth	0.055	1.065	.488
Condition	0.186	0.567	.022
PSQI Change Score (Overall Sleep)	0.299	0.101	.000

^a^Betas are standardized linear regression coefficients

^b^Sleep question removed from PHQ-9

**Table 3:** Change in Sleep Subscale Scores from Six Weeks to Seven Months Postpartum Predicting Seven Month Depressive Symptoms

Variable	Beta^a^	SE	*P*-value
Baseline PHQ-9 (Depressive Symptoms)^b^	0.418	0.082	.000
Breastfed at Birth	0.067	1.033	.381
Condition	0.189	0.548	.016
PSQI Change Score -Daytime Dysfunction	0.370	0.308	.000
Baseline PHQ-9 (Depressive Symptoms)^b^	0.341	0.084	.000
Breastfed at Birth	0.042	1.075	.595
Condition	0.178	0.573	.030
PSQI Change Score -Sleep Latency	0.279	0.409	.001
Baseline PHQ-9 (Depressive Symptoms)^b^	0.376	0.081	.000
Breastfed at Birth	0.078	1.046	.317
Condition	0.214	0.554	.007
PSQI Change Score -Sleep Quality	0.345	0.496	.000

^a^Betas are standardized linear regression coefficients

^b^Sleep question removed from PHQ-9

## Discussion

Several studies have examined the relationship between poor sleep and postpartum depression [13]. However, some studies have examined the effect of sleep on postpartum depression at less than four weeks [18–20] and many studies have examined sleep and postpartum depression at the same time point [21–26]. Therefore, it is difficult to make potential causal inferences about the effect of sleep on postpartum depression. Sleep disturbances could lead to an increase in depressive symptoms; however, it is equally plausible that depressive symptoms lead to disrupted sleep. It is difficult to identify the direction of the effect when sleep problems and depressive symptoms are examined at the same time point. Our study addressed this limitation by assessing changes in self-reported sleep from six weeks to seven months postpartum. Additionally, little is known regarding the effect of sleep changes on depressive symptoms later during the postpartum phase among women who are at heightened risk for postpartum depression.

Consistent with previous studies [13], worsening of self-reported sleep issues from six weeks to seven months postpartum was related to higher depressive symptoms at seven months. This is consistent with Okun and colleagues [30] who found that poor sleep was predictive of postpartum depression during the first 17 weeks postpartum. Based on our findings, it is important for interventions to address sleep problems early in the postpartum phase and to prevent sleep problems from worsening.

Results also indicated that changes in self-reported sleep latency (amount of time it takes to fall asleep at night), difficulty staying awake during the day, and sleep quality were related to depressive symptoms at seven months. Therefore, it is important for practitioners to monitor these sleep problems over time. Practitioners should discuss with their patients at the six-week appointment what types of sleep changes to watch for in order to prevent depressive symptoms. Potential prevention strategies that could be discussed include feeding the baby more often during the day, keeping the baby close in the evening to encourage cluster feeding before bed, and going to bed immediately after feeding the baby at night. Additionally, good sleep hygiene and habits should be taught that could include naps that are short in duration, limiting light exposure during the night, avoiding caffeine after a certain time, relaxation exercises, and cognitive restructuring to address worrying [44].

Participants reported higher scores on the PSQI (indicating poorer sleep) than what has been reported in the literature among adults [31]. For example, one study found that normal adults scored on average 2.67; however, our participants reported an average of 7.11 at six weeks postpartum and 5.53 at seven months postpartum [31]. This was expected given the disruptive nature of postpartum sleep. For example, one study found that women who were between four to eight weeks postpartum woke up on average three times per night and were awake for 49 min each awakening [45]. It has been hypothesized that postpartum depression could be the result of imbalances in hormones [46, 47]. However, it is equally plausible that poor sleep could be a significant contributor to postpartum depression.

### Limitations

There were several limitations of this study. First, we did not objectively measure sleep using an objective device such as an ActiGraph. Therefore, this study relied only on self-report. Second, despite examining sleep and depressive symptoms at different time points, we are still not able to make causal inferences given the lack of randomization. Furthermore, infant bedsharing was not assessed, which could have influenced maternal sleep and been a confounding variable in our study. Third, our sample included mostly Caucasian, highly educated, high income women and it is uncertain how our findings would generalize to a more diverse sample. Fourth, we examined depressive symptoms rather than diagnosed depression as our primary dependent variable. However, we believe it is important to examine depressive symptoms given many women experience some depressive symptoms without meeting the full criteria for depression [12]. Fifth, in the overall study, participants had been randomized to an exercise or control condition, which may have influenced the results. However, we controlled for condition assignment in our regression models and both arms exercised at similar rates so it is unlikely that the condition assignment influenced the results. Finally, depressive symptoms were only assessed at six weeks and seven months postpartum. This is problematic given it is possible that the depressive symptoms fluctuated between the assessment sessions.

## Conclusions

Additional research is needed that examines the relationship between sleep and postpartum depressive symptoms. Future studies should use actigraphy to evaluate sleep, and examine both sleep and depressive symptoms at multiple intervals throughout the first postpartum year. Additional sleep and depression studies are needed among diverse populations. For example, 38% of low income women experience postpartum depression, which is 2–3 times more likely to occur in this population than the general population of postpartum women [48]. Therefore, this population of women is especially important to examine. Finally, intervention studies are needed that target sleep hygiene and habits in order to prevent postpartum depressive symptoms among women at risk. For example, future studies could examine the efficacy of cognitive –behavioral therapy for insomnia among women who are at high risk for postpartum depression (e.g., history of depression prior to pregnancy) and experiencing sleep disturbances. It would be interesting to determine if therapy focused on sleep hygiene, sleep habits, cognitive restructuring, and relaxation for example would influence the quantity and quality of sleep and consequently, reduce the risk of postpartum depression.

Policy makers and practitioners should be made aware of the relationship between poor sleep and higher depressive symptoms. Women typically attend a six week postpartum provider visit and both sleep and depressive symptoms should be assessed at this visit. Even though the patient may not meet the criteria for depression, reporting poor sleep at this appointment could be a warning sign for depressive symptoms in the future. The practitioner should discuss with the patient strategies for improving sleep (e.g., sleep when the baby sleeps, go to bed immediately after feeding the baby at night, or behavioral sleep intervention for the infant); [49] and how to prevent sleep quality from worsening during the first postpartum year. There are also environmental and other health-related factors that can influence sleep such as caffeine consumption, uncomfortable sleeping arrangements (e.g., noise, darkness), physical symptoms such as Nocturia (i.e., frequent urination during the night), and electronic media use before bed, which should be addressed in addition to infant-related sleep disruption. Taken together, both policy makers and practitioners have the potential to make a significant impact on improving sleep and consequently depression among postpartum women.

## Abbreviations

PHQ-9: Patient Health Quesitonnaire-9
PSQI: Pittsburgh Sleep Quality Index
